# Identification of markers correlating with mitochondrial function in myocardial infarction by bioinformatics

**DOI:** 10.1371/journal.pone.0316463

**Published:** 2024-12-30

**Authors:** Wenlong Kuang, Jianwu Huang, Yulu Yang, Yuhua Liao, Zihua Zhou, Qian Liu, Hailang Wu

**Affiliations:** 1 Department of Cardiology, Traditional Chinese and Western Medicine Hospital of Wuhan, Tongji Medical College, Huazhong University of Science and Technology, Wuhan, Hubei, China; 2 Department of Cardiology, Wuhan No.1 Hospital, Wuhan, Hubei, China; 3 Department of Cardiology, Union Hospital, Tongji Medical College, Huazhong University of Science and Technology, Wuhan, China; 4 Hubei Key Laboratory of Biological Targeted Therapy, Union Hospital, Tongji Medical College, Huazhong University of Science and Technology, Wuhan, China; 5 Hubei Engineering Research Center of Immunological Diagnosis and Therapy of Cardiovascular Diseases, Union Hospital, Tongji Medical College, Huazhong University of Science and Technology, Wuhan, China; 6 Center for Reproductive Medicine, Wuhan Children’s Hospital, Tongji Medical College, Huazhong University of Science and Technology, Wuhan, China; Rutgers: Rutgers The State University of New Jersey, UNITED STATES OF AMERICA

## Abstract

**Background:**

Myocardial infarction (MI), one of the most serious cardiovascular diseases, is also affected by altered mitochondrial metabolism and immune status, but their crosstalk is poorly understood. In this paper, we use bioinformatics to explore key targets associated with mitochondrial metabolic function in MI.

**Methods:**

The datasets (GSE775, GSE183272 and GSE236374) were from National Center for Biotechnology Information (NCBI) Gene Expression Omnibus (GEO) in conjunction with mitochondrial gene data that were downloaded from the MitoCarta 3.0 database. Differentially expressed genes (DEGs) in the dataset were screened by ClusterGVis, Weighted Gene Co-Expression Network Analysis (WGCNA) and GEO2R, and functional enrichment was performed by Gene Set Enrichment Analysis (GSEA) and Kyoto Encyclopedia of Genomes (KEGG). Then mitochondria-associated DEGs (MitoDEGs) were obtained. Protein-protein interaction (PPI) networks were constructed to identify central MitoDEGs that are strongly associated with MI. The Cytoscape and miRWalk databases were then used to predict the transcription factors and target miRNAs of the central MitoDEG, respectively. Finally, the mouse model has been established to demonstrate the expression of MitoDEGs and their association with cardiac function.

**Results:**

MitoDEGs in MI were mainly involved in mitochondrial function and adenosine triphosphate (ATP) synthesis pathways. The 10 MI-related hub MitoDEGs were then obtained by eight different algorithms. Immunoassays showed a significant increase in monocyte macrophage and T cell infiltration. According to animal experiments, the expression trends of the four hub MitoDEGs (Aco2, Atp5a1, Ndufs3, and Ndufv1) were verified to be consistent with the bioinformatics results.

**Conclusion:**

Our study identified key genes (Aco2, Atp5a1, Ndufs3, and Ndufv1) associated with mitochondrial function in myocardial infarction.

## 1. Introduction

Myocardial infarction is one of the most prevalent diseases in the world in terms of morbidity and mortality, with its mechanism being primarily obstruction of coronary arteries leading to myocardial cell injury and causing a widespread inflammatory response [1]. Timely and effective reperfusion therapy can restore blood circulation to avoid excessive destruction of cardiac function. However, the therapeutic effect of reperfusion remains unsatisfactory due to the fact that myocardial infarction is pathologically multifactorial [2]. Further physiological mechanisms need to be explored.

The inflammatory phase after MI is an important stage in the transition to the repair phase, and excessive inflammation will lead to poor repair of the cardiac [3]. Endogenous signals released by cardiomyocytes will cause a burst of cytokines and chemokines, a variety of immune cells will migrate and infiltrate the area of infarction, while the production of reactive oxygen species (ROS) further amplifies the inflammatory response [4]. Mitochondria are the main site of ROS production, as demonstrated in some studies showing that mitochondrial dysfunction after MI is an important cause of cardiac function decline [5]. Mitochondria are in high enrichment in myocardial tissues and play an important role in ATP generation and signaling during metabolic activities in cardiomyocytes [6]. However, disruption of blood supply from MI or IR will result in microvascular damage, ischemia-reperfusion (IR) bursts, apoptosis, and damage to mitochondria [7]. The function of mitochondrial respiratory chain enzymes is affected and deteriorated during hypoxia in cardiomyocytes, leading to the formation of superoxide and hydrogen peroxide [8].

Mitochondria have an important role in the regulation of immune cells related to activation, differentiation and survival [9]. Major processes in which mitochondria are involved, such as amino acid metabolism, antioxidant pathways, mitochondrial autophagy, and mitochondrial ROS production (mtROS), are inextricably linked to immune function [10]. For example, the mitochondrial uncoupling protein 2 (UCP2) controls the production of ROS and affects macrophage polarization, and tricarboxylic acid cycle (TCA) in M1 and M2 macrophage has different profiles [11, 12]. After MI, disruption of mitochondrial function or structure in the cardiac will result in an imbalance in the mitochondrial quality control system, impaired mitochondrial dynamics, and excessive mitochondrial fission or fusion of mitochondria that will impair oxidative phosphorylation (OXPHOS) processes and thus reduce ATP production [13], macrophage switching to M1 type and amplification of downstream inflammatory response, which is detrimental to cardiac repair [14]. While ROS, generated by mitochondrial metabolic imbalance, contribute to Nod-like receptor (NLR) family pyrin domain containing 3 (NLRP3) inflammasome vesicle activation, leading to the formation of pro-inflammatory factors interleukin-1β (IL-1β) and interleukin-18 (IL-18) [15]. Mitochondrial ROS affect T cell activity through mitochondrial glycerol-3-phosphate dehydrogenase 2 (GPD2) production and also alter hemoglobin to regulate B cell function [16, 17]. Imbalances in mitochondrial metabolic function and altered immune cell status are both associated with cardiac remodeling after myocardial infarction [14], but their crosstalk has not been reported and requires further investigation.

In view of the complexity and importance of mitochondria in the pathogenesis of MI, the identification of this key process will be beneficial in revealing potential therapeutic targets for MI. In addition to the mRNAs we are concerned with, the microRNAs have now attracted more attention in MI and may be related to emergency response [18]. Multiple miRNAs have shown potential promise as biomarkers and therapeutic targets in MI [19–21]. Mitochondria-associated miRNAs also play a very important role in regulating mitochondrial homeostasis, and therefore the discovery of new mitochondria-associated mRNA or miRNA markers is critical for MI. Bioinformatics is an essential tool for exploring potential target molecules for diseases as well as therapeutic approaches. Based on relevant microarray data from the GEO database, this study explored whether and how mitochondria-related genes contribute to the development of myocardial infarction. Additionally, genes associated with mitochondrial function in myocardial infarction were also investigated to provide a better understanding of potential targets in myocardial infarction.

## 2. Methods

### 2.1 Microarray data retrieval

Datasets were obtained from the public repository NCBI GEO (http://www.ncbi.nlm.nih.gov/ geo) [22] using "myocardial infarction" as the search queries. We screened them further based on information such as sequencing type (transcriptology), animal species (Mus musculus), sample source (ventricle), and modeling time. Finally, GSE775 was obtained. The GSE775 ([MG_U74Av2] Affymetrix Murine Genome U74A Version 2 Array) is generated by the GEO platforms 81 (GPL81) platform that contains 59 left ventricular (LV) samples from mus musculus. In order to have a better analysis of the DEGs between the MI group and the control (CON) group, three myocardial infarction hearts were collected for analysis at different time points after MI, including 1 hour, 4 hours, 24 hours, 48 hours, 1 week, and 2 weeks after MI [23]. The GSE236374 {Illumina NovaSeq 6000 (Mus musculus)} is generated by GPL24247 platform and composed of 9 LV samples from MI mice (n = 6) and CON mice (n = 3), the mice were eight-week-old males [24]. The GSE183272 {Illumina NovaSeq 6000 (Mus musculus)} is generated by GPL24247 platform and composed of 30 LV samples from MI mice (n = 15) and CON mice (n = 15).

### 2.2 Gene expression profiles

Data of each microarray were accessed from GEO using R package "GEO query". DEGs in each microarray were obtained by using the R package "limma" as implemented by GEO2R online tool (https://www.ncbi.nlm.nih.gov/geo/geo2r/) [22], we normalized and corrected all gene expression profiling microarray data and annotated gene names using the limma package (version 3.44.0) for R (version 4.0.2). We used the SVA package (version 3.36.0) to remove batch effects, and all identified DEGs met p < 0.05 and |log2 (Foldchange)|≥ 1. Visualization of the generated DEG by Volcano Plot using the R package "ggplot2" [25] and Heat-map using R package "ComplexHeatmap" [26]. To cluster and enrich the trend of DEGs, the ClusterGVis (https://github.com/junjunlab/ClusterGVis) [27] and ClusterProfiler package were employed [28].

### 2.3 Functional enrichment analysis

R package "clusterProfiler" [28] was used to complete the enrichment analysis, with the "c2.cp.v7.2.symbols.gmt" (https://www.gsea-msigdb.org/gsea/msigdb/index.jsp) as the reference gene set with 10,000 alignments and a significance threshold of 10. Visualization of results using the R package "ggplot2" [25]. The DEG was analyzed for Gene Ontology (GO) and KEGG pathway enrichment using the R package "clusterProfiler", and the items with P < 0.05 in BenjaminiHochberg test were considered to be statistically significant. Results were visualized by chordal and circular plots using the R packages "ggplot2" and "GOplot" [29].

### 2.4 Identification of mitochondria‑related DEGs (MitoDEGs)

1,140 mitochondria-localized genes were obtained by accessing the mitochondrial protein database, MitoCarta3.0 (http://www.broadinstitute.org/mitocarta) [30]. Mitochondrial DEGs were obtained by intersecting the DEGs in each microarray with 1,140 mitochondrial locus genes using Venn Diagram and visualized as heatmaps using the R package "ggplot2" [25]. Four overlapping MitoDEGs in microarrays were ultimately acquired.

### 2.5 Protein-protein interaction (PPI) analysis and hub gene identification

The PPI was generated by the STRING (v12.0) biological database (https://string-db.org/, accessed on 26 July 2023). To construct the PPI, interaction scores of>0.4 were selected, and disconnected nodes were deleted. For species we have chosen Mus musculus. Next, the results generated were visualized as a network using Cytoscape software version 3.7.1 [31]. The Cyto-Hubba plug-in and MCODE plug-in in Cytoscape software were used to find the hub genes in the PPI network [32, 33]. The Cytoscape plugin cytoHubba—with algorithms including Maximum Clique Centrality (MCC), Closeness, Maximum Neighborhood Component (MNC), Degree, Edge Percolated Component (EPC), Betweenness, Radiality and Stress was used to predict the hub genes, and used a Upset plot to visualize the final results [32].

### 2.6 Weighted gene co-expression network analysis

To identify co-expression modules, we used the WGCNA package in R (version 1.71) to create unsigned co-expression networks [34]. First, we performed cluster analysis and fit index analysis on all samples, converting the similarity matrix into an adjacency matrix, then calculated the optimal efficacy (soft threshold) value to ensure that the correlation between connectivity and efficacy was higher than 0.9. The power value was set at 4. Based on this, we constructed the scale-free network and the topological overlap matrix (TOM). A corresponding TOM dissimilarity analysis (diss TOM) was also performed, and finally a genetically (dendrogrammed) hierarchical clustering tree based on the hierarchical clustering function hclust was generated for module detection.

### 2.7 The prediction of MitoDEGs-miRNAs central network

For the purpose of exploring the regulatory factors upstream of central MitoDEG, TFs of central MitoDEG were predicted using iRegulon, a plugin for Cytoscape 3.8.2 [35], and the miRNAs of central MitoDEG were predicted with the miRWalk database (http://mirwalk.umm.uni-heidelberg.de) [36]. The hub MitoDEG, the obtained TFs and miRNAs were visualized as networks by Cytoscape 3.7.1.

### 2.8 Immune infiltration analysis

Considering the heterogeneity of stromal and immune cell infiltration in human and mouse, we used the mMCP-counter software package to evaluate stromal and immune cell infiltration in the combined RNA-seq dataset of GSE236374 and GSE183272 [37].

### 2.9 Construction of animal models with MI

Male C57BL/6N mice aged 5 weeks were purchased from Charles River Laboratories and maintained at the Laboratory Animal Centre of Huazhong University of Science and Technology, approval number No. 422023600011180. Male C57BL/6 mice were randomly divided into two groups: 1) Control group (CON) (n = 6); 2) Myocardial infarction group (MI) (n = 6); The MI group was induced by permanent ligation of the left anterior descending branch of the coronary artery. One week later, we performed an echocardiogram on the mice, observing the ejection fraction, the degree of dilatation of the left ventricle, and the thickness of the ventricular wall to determine cardiac function. All mice were sacrificed on day 7 for histopathological examinations. In order to minimize the stress and pain of the mice, our experimenters are trained and tested to euthanize the mice by cervical dislocation to minimize the pain of the mice.

### 2.10 Total RNA isolation and quantitative reverse transcription-polymerase chain reaction

Total RNA was extracted from mouse heart tissue by an RNA isolator (Vazyme, Nanjing, China) and subjected to reversely transcribed into cDNA using HiScript^®^ III qRT SuperMix (Vazyme, Nanjing, China). Subsequently, real-time Polymerase Chain Reaction (PCR) was performed with SYBR Green Master Mix (Vazyme, Nanjing, China) on a CFX Manager Software (Bio-Rad) following the manufacturer’s instructions. The 2^−ΔΔCt^ relative quantification method, using glyceraldehyde-3-phosphate dehydrogenase (GAPDH) for normalization, was used to estimate the amount of target mRNA in samples, and fold ratios were calculated relative to mRNA expression levels from control samples [38].

### 2.11 Western blotting

Following weighing, frozen tissues were homogenized by lysis in ice-cold modified RIPA buffer containing protease and phosphatase inhibitors (Beyotime, Shang-hai, China). Then, the homogenate was centrifuged and the protein concentration of the supernatant was determined using a Bradford protein assay kit (Thermo Fisher Scientific, Mas-sachusetts, USA). Twenty-five micrograms of protein from heart tissue were added to a 15-μL protein loading system. Proteins were separated by 10%- or 12.5%-PAGE gels (Yamei, Shanghai, China) and transferred to a polyvinylidene fluoride (PVDF) membrane (Millipore, Massachusetts, USA). The blots were probed with the following primary antibodies for a 12-h incubation: anti-Aconitase 2 (Aco2) monoclonal antibody (1:1000, abclonal, Wuhan, Hubei), anti-ATP synthase F1 subunit alpha (Atp5a1) (1:500, abclonal, Wuhan, Hubei), anti- NADH: ubiquinone oxidoreductase core subunit S3 (Ndufs3) (1:1000, abclonal, Wuhan, Hubei)), anti- NADH: ubiquinone oxidoreductase core subunit V1 (Ndufv1) (1:1000, Abcam, abclonal, Wuhan, Hubei)), anti-GAPDH monoclonal antibody (1:3000, Protein-tech, Wuhan, Hubei) and anti-a-Tubulin monoclonal antibody (1:3000, Protein-tech, Wuhan, Hubei). The Western blotting results were quantified using Image Lab software (Bio-Rad Laboratories, CA, USA) and were expressed normalized to the mean of the control group.

### 2.12 Echocardiography and Doppler imaging

Transthoracic echocardiography was performed using a VisualSonics Vevo 3100 system equipped with an MX400 transducer (Visual Sonics, Toronto, Canada). Left ventricular ejection fraction (LVEF) and other indices of systolic function were obtained from a short-axis M-mode scan at the midventricular level, as indicated by the presence of papillary muscles, in conscious, gently restrained mice. Apical 4-chamber views were obtained in anesthetized mice for diastolic function measurements using pulsed-wave Doppler imaging at the level of the mitral valve. Anesthesia was induced by 5% isoflurane and confirmed by a lack of response to firm pressure on one of the hind paws. During echocardiogram acquisition, isoflurane was reduced to 1.0–1.5% and adjusted to maintain a heart rate in the range of 500–650 beats per min under body-temperature-controlled conditions. At the end of the procedures, all mice recovered from anesthesia without difficulties. All parameters of left ventricular (LV) structure and function were measured at least three times, and the means are presented.

### 2.13 Statistical analysis

All data are expressed as means ± SD. Student’s t-test (for comparisons between two groups) was used for the statistical analysis using IBM SPSS Statistics version 25, P<0.05 was consider as significant. The calculations were performed using Prism 6.0 (GraphPad Software, La Jolla, California, USA).

## 3. Results

### 3.1 DEGs in MI and functional enrichment analysis by ClusterGVis and WGCNA

**Fig 1:**
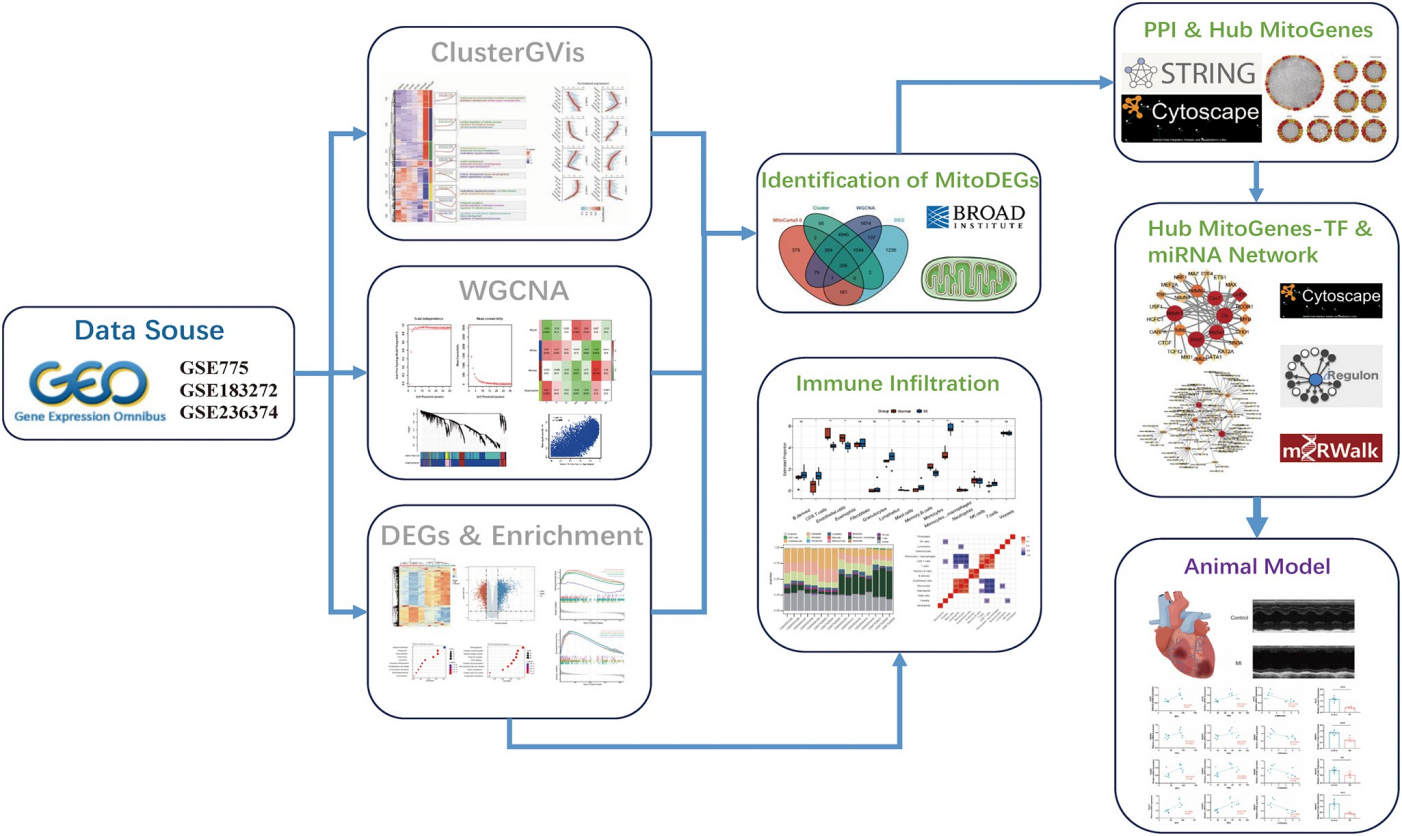
Flowchart of the multistep screening strategy on bioinformatics data.

**Fig 2:**
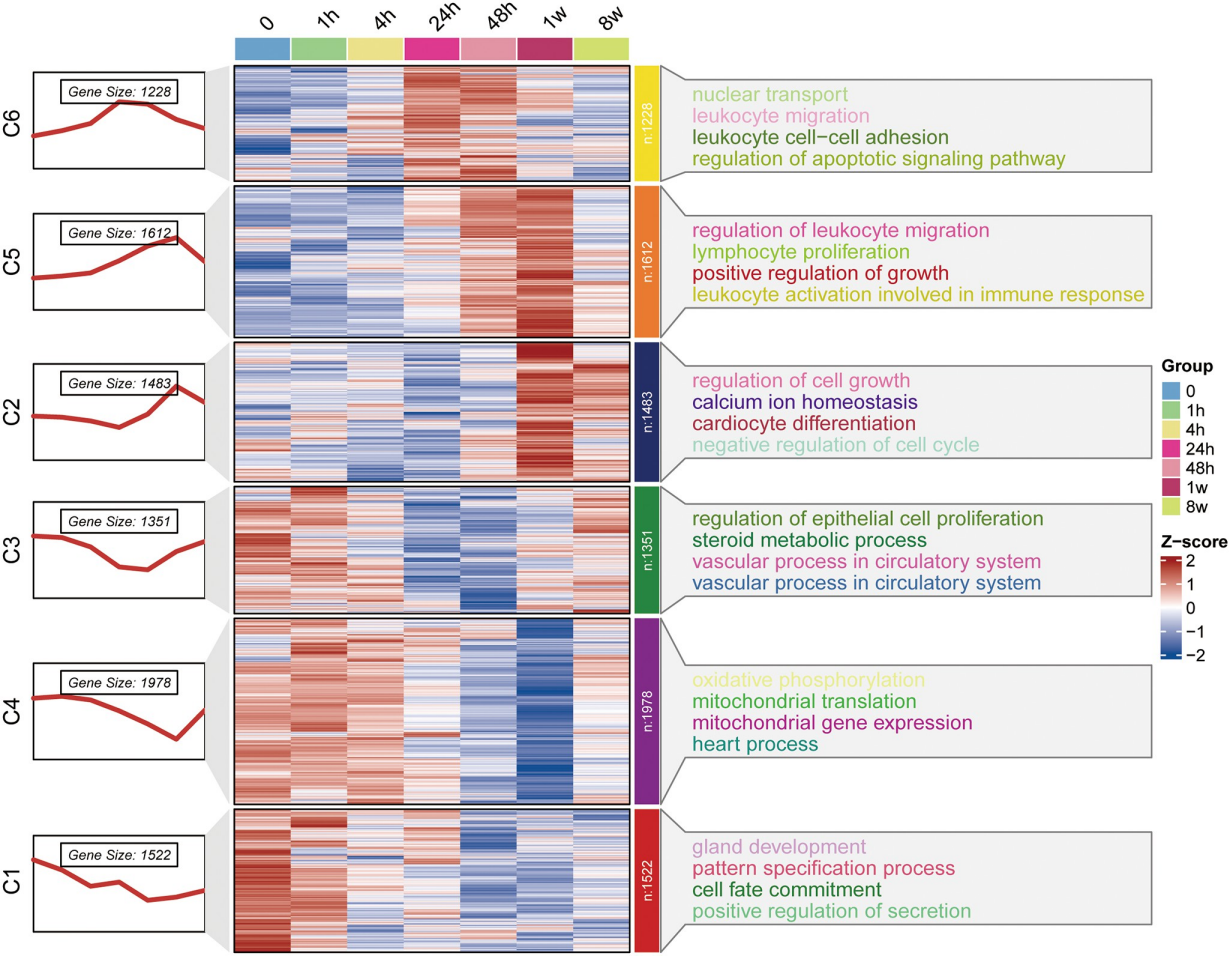
Differential gene analysis and functional enrichment of the GSE775 dataset over time using the ClusterGVis package. **C1-C6**: The left side shows a line plot of the expression trend of DEGs in each cluster of the all samples. The left side of the rectangle shows the serial number of clusters, and the number of genes within each cluster (cluster size) is shown below the line plot. In the middle is a heat map of the clusters of DEGs in the control and MI samples. The right side shows the pathways that were significantly enriched for DEGs in each cluster after KEGG enrichment analysis.

**Fig 3:**
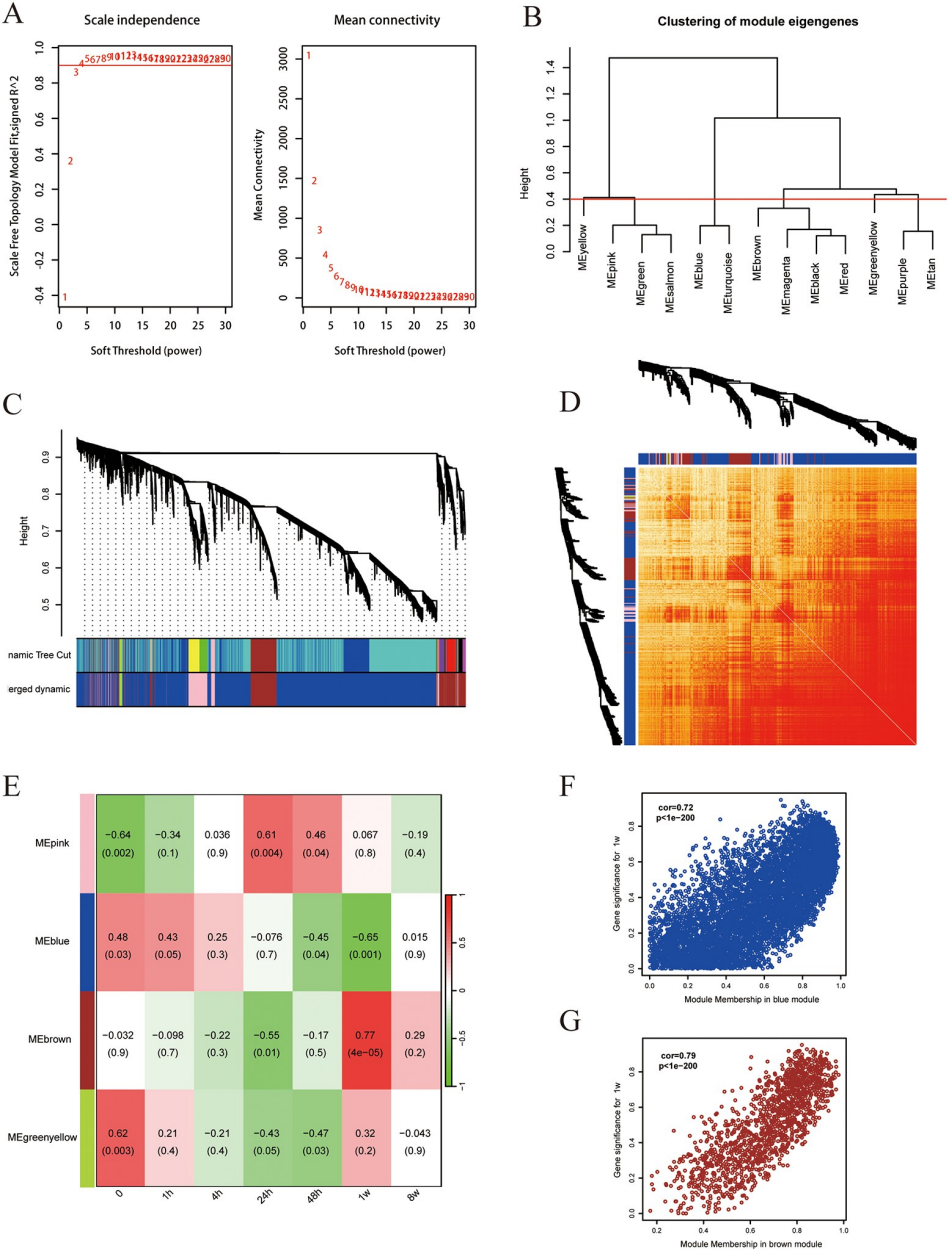
Construction of weighted co-expression network related datasets and identification of related key modules. **A**. Network topology analysis for various soft thresholds(β); **B**. Clustering of block feature genes. A cut line (0.4) was selected for the module dendrogram and a number of modules were merged based on the estimated similarity of the module feature genes; **C**. Gene dendrograms were obtained by average chained hierarchical clustering. The colored rows below the dendrogram show the module assignments determined by the dynamic tree-cutting method; **D**. The heatmap shows the Topology Overlap Matrix (TOM) values between the proteins of the modular network divided with the dynamic method. Yellow represents low topological overlap matrix values and red represents high topological overlap matrix values; **E**. WGCNA Module Adjacency Heat Map; **F-G**. Scatter plots of the degree of Cox regression and P-value in the dataset. x-axis represents the degree of regression and y-axis represents gene significance. Each circle represents a gene.

The whole data filtering strategy is shown in Fig 1. We identified the key genes in the three datasets by various methods such as ClusterGVis and WGCNA and performed enrichment analysis. After taking the intersection with mitochondria-related genes, we obtained the MitoDEGs and further performed miRNA prediction and immune infiltration analyses to study the relationship between the MitoDEGs and immune cells, and finally constructed the animal model for validation. The dataset GSE775 was selected, and the time series of gene expression was firstly clustered and visualized by the ClusterGVis package, and the differential analysis was carried out at the time of infarction, and at 1h, 4h, 24h, 48h, 1w and 2w post-infarction, respectively. The results showed that the differences of the expressed genes were the most significant at 1w post-infarction, and through the functional annotations, the DEGs were mainly involved in positive regulation of growth, cardiocyte differentiation, vascular process in circulatory system, mitochondrial gene expression and pattern specification process (Fig 2). Thereafter, we analyzed the GSE775 dataset using WGCNA in order to first find the optimal soft thresholds and draw gene clustering trees (Fig 3A and 3B), followed by clustering trees and correlation heatmaps between modules, also among the different time points (Fig 3C–3E). By analyzing the correlation between each gene module and phenotype (different periods of infarction), we found that the blue module (cor = 0.72, P < 1e-200) and the brown module (cor = 0.79, P < 1e-200) showed the most representative features (Fig 3F and 3G).

### 3.2 Merging datasets-DEGs in MI and functional enrichment analysis

**Fig 4:**
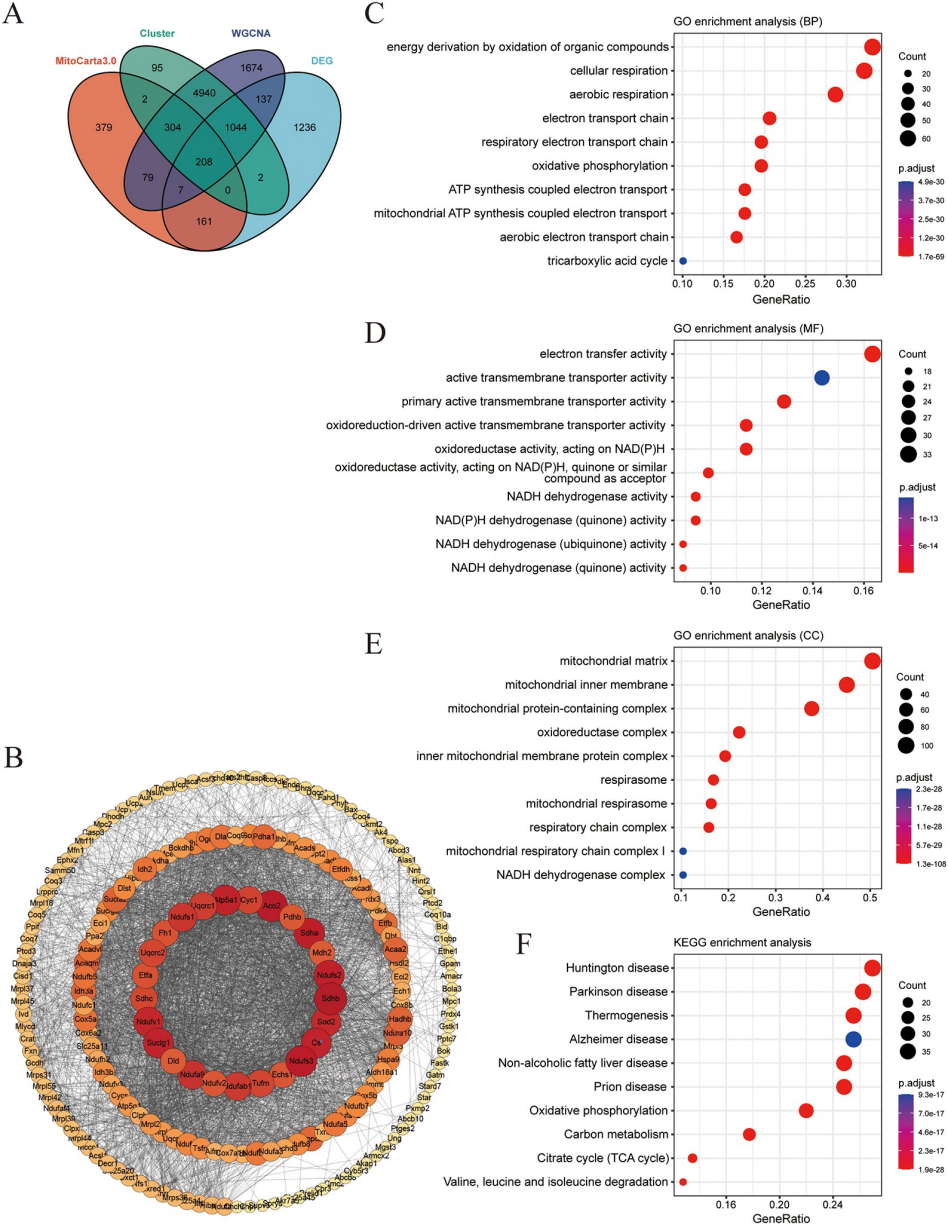
MitoDEGs in DCM. **A**. Venn diagrams showed the number of Hub MitoDEGs; **B**. PPI network. The circles in the PPI network represent proteins and the lines represent interactions between proteins. The red depth indicates the level of importance; **C-E.** The enriched BP, GO, CC terms of up-regulated DEGs in Hub MitoDEGs; **F**. KEGG pathway enrichment results in Hub MitoDEGs.

On the basis of the previous analyses, we selected the datasets GSE236374, GSE183272 from mice 1 week after infarction, and then combined and removed batch effects for both and visualized them with PCA plots. Fig 4A and 4B show the PCA analysis of GSE236374 and GSE183272 before and after de-batching, and it can be found that the batch effect was obviously removed (S1A and S1B Fig). Whereas Fig 4C and 4D show the PCA analyses of the control and MI groups before and after de-batching, it can be noticed that no differences between the samples were removed (S1C and S1D Fig). Analysis of variance showed that there were 2817 DEGs in the GSE236374 dataset, including 1758 up-regulated genes and 1059 down-regulated genes, as well as 2359 DEGs in the GSE183272 dataset, including 1410 up-regulated genes and 949 down-regulated genes compared with the control samples. The DEGs were displayed as Volcano Plots and Heatmaps (S1E and S1F Fig).

Functional enrichment for GO and KEGG pathway analyses was further processed for up-regulated DEGs and down-regulated DEGs, respectively. The most enriched GO terms were categorized as Biological Process (BP), Cellular Component (CC) and Molecular Function (MF). Among the genes up-regulated in expression, the main topics included leukocyte-mediated immunity, leukocyte migration; actin binding, cell adhesion molecule binding; collagen-containing extracellular matrix, actin cytoskeleton. The KEGG pathway enriched in DEGs was mainly involved in cytokine-cytokine receptor interactions, phagolysosomes (S2A–S2D Fig). And the genes down-regulated in expression, the main topics included precursor metabolites and energy production; channel activity, passive transmembrane transport protein activity; mitochondrial protein-containing complexes, mitochondrial matrix. The KEGG pathway enriched in DEGs was involved in oxidative phosphorylation. channel activity, passive transmembrane transporter protein activity; mitochondrial protein-containing complexes, mitochondrial matrix. KEGG pathways enriched in DEGs are involved in oxidative phosphorylation (S2E–S2H Fig).

GSEA shows that the two datasets merge and are then primarily involved in metabolic cycles as well as immune-related pathways, including carbon metabolism, TCA, cell adhesion molecules, cytokine signaling pathway. The signaling pathways involved are NF-kappa B signaling pathway, NOD-like receptor signaling pathway, PI3K-Akt signaling pathway. In addition, it is shown that participation in antigen processing and presentation, cytokine-cytokine receptor interaction, natural killer cell mediated cytotoxicity (S3A–S3D Fig).

### 3.3 MitoDEGs in MI

Mitochondria-related genes were retrieved from the MitoCarta3.0 database, and choose to take the intersection with the DEGS of the merged two datasets and the DEGS obtained from the analysis of GSE775 using both ClusterGVis and WGCNA methods (Fig 4A). Finally, we obtained 208 DEGs. PPIs of 208 MitoDEGs were analyzed using the STRING database and visualized as a network using Cytoscape. The most significant DEGs are shown in the figure and include Ndufs1, Atp5a1, Aco2, Sdha, Ndufs2, Sdhb, Sod2, Cs, Ndufs3, and more (Fig 4B). The central DEGs were then enriched by GO and KEGG analyzes. The results indicate that energy derivation by oxidation of organic compounds, cellular respiration, aerobic respiration (BP), electron transfer activity, active transmembrane transporter activity (MF), mitochondrial matrix, mitochondrial inner membrane, Mitochondrial protein-containing complex (CC), KEGG results showed enrichment into signaling pathways including Thermogenesis, Oxidative phosphorylation, carbon metabolism, TCA (Fig 4C–4F).

**Fig 5:**
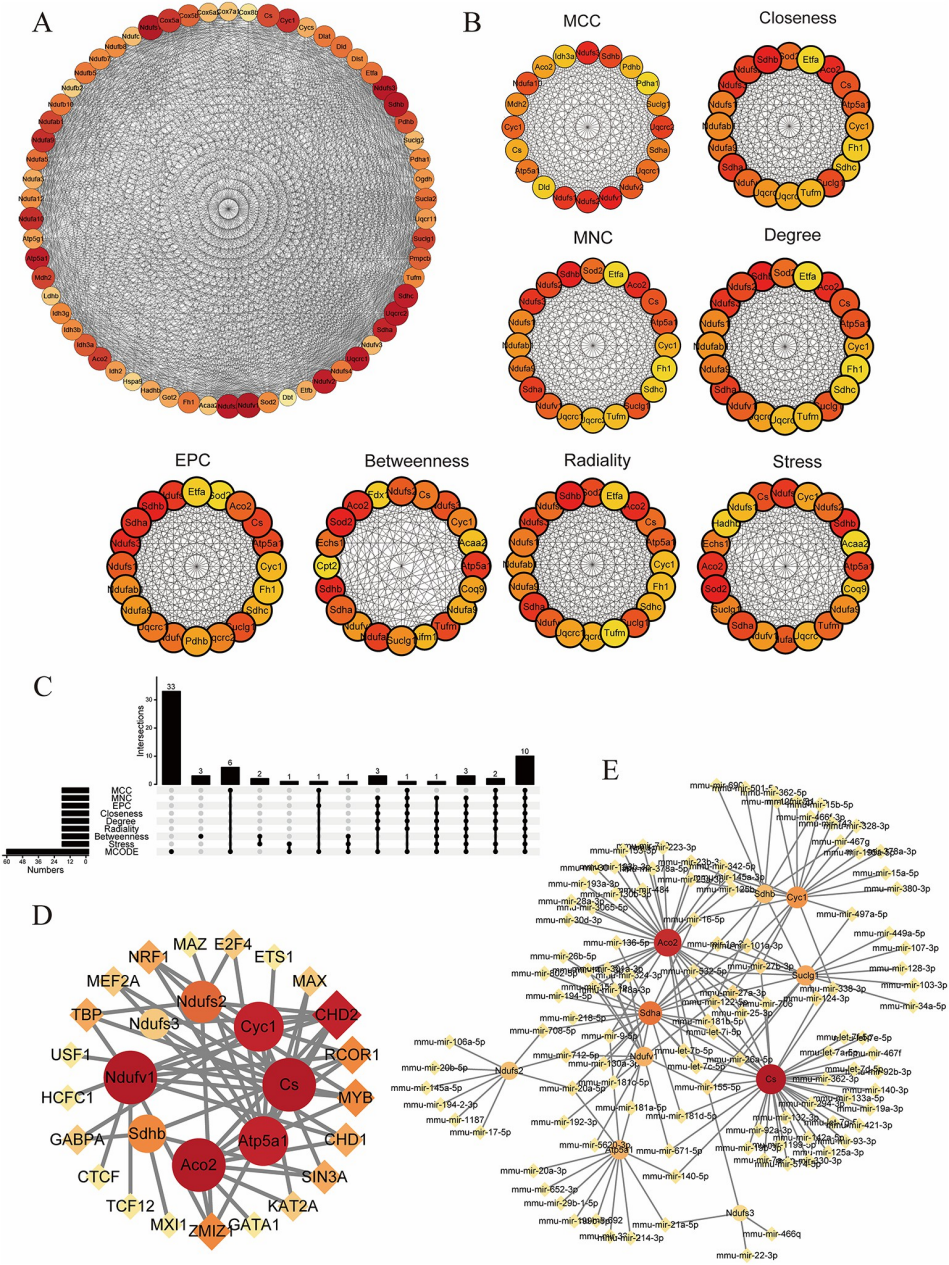
MitoDEGs in MI; PPI network analysis and hub MitoDEGs identification. Hub MitoDEGs-TFs-miRNAs regulatory network; A. Biology module from cytoscape’s MCODE plug-in; B. Hub gene identification using eight Cytohubba plugins (MCC, Closeness, MNC, Degree, EPC, Betweenness, Radiality and Stress) in Cytoscape; C. Upset Venn diagram applied to demonstrate the predicted hub genes; D. TF–hub MitoDEGs regulatory network: the red squares represent hub MitoDEGs, and the yellow dots represent transcription factors; E. miRNA–hub MitoDEGs regulatory network: the red squares represent hub MitoDEGs, and the yellow dots represent miRNA.

Next, we used the MNN, DMNC, MNC, Degree, EPC, BottleNeck, Radiality, and Stress algorithms in Cytoscape software to analyze the genes again with the PPI interactions network to identify the hub genes, and the combination of the analyses finally resulted in eight hub mitoDEGs, including Ndufv1, Ndufs2, Ndufs3, Cyc1, Cs, Atp5a1, Aco2 and Sdhb (Fig 5A–5C).

### 3.4 Hub MitoDEGs‑TFs‑miRNAs regulatory network

We explored the upstream regulation of central MitoDEGs by predicting associated transcription factors and miRNAs. The transcription factors of hub MitoDEGs were predicted by using the iRegulon plugin in Cytoscape software, and a transcription factor regulatory network for hub MitoDEGs was constructed consisting of 21 transcription factors (MAZ, E2F4, ETS1, MAX, CHD2, RCOR1, MYB, CHD1, SIN3A, KAT2A, GATA1, ZMIZ1, MXI1, TGF12, CTCF, GABPA, HCFC1, USF1, TBP, MEF2A, NRF1) (Fig 5D). MiRNAs of hub MitoDEGs were predicted using miRWalk 3.0 software, and a regulatory network of hub MitoDEGs-miRNAs containing 125 nodes and 174 edges was constructed. We identified four miRNAs, of which miR-27a-3p and miR-27b-3p both interacted with Cs, Aco2, Sdh2, Cyc1, and Suclg1. While miR-122-5p interacted with Cs, Aco2, Ndufv1, and Sdhb, and finally miR-155-5p had interaction with Cs, Suclg1, Ndufv1, and Ndufs3 (Fig 5E).

### 3.5 Immune cell infiltration in MI

**Fig 6:**
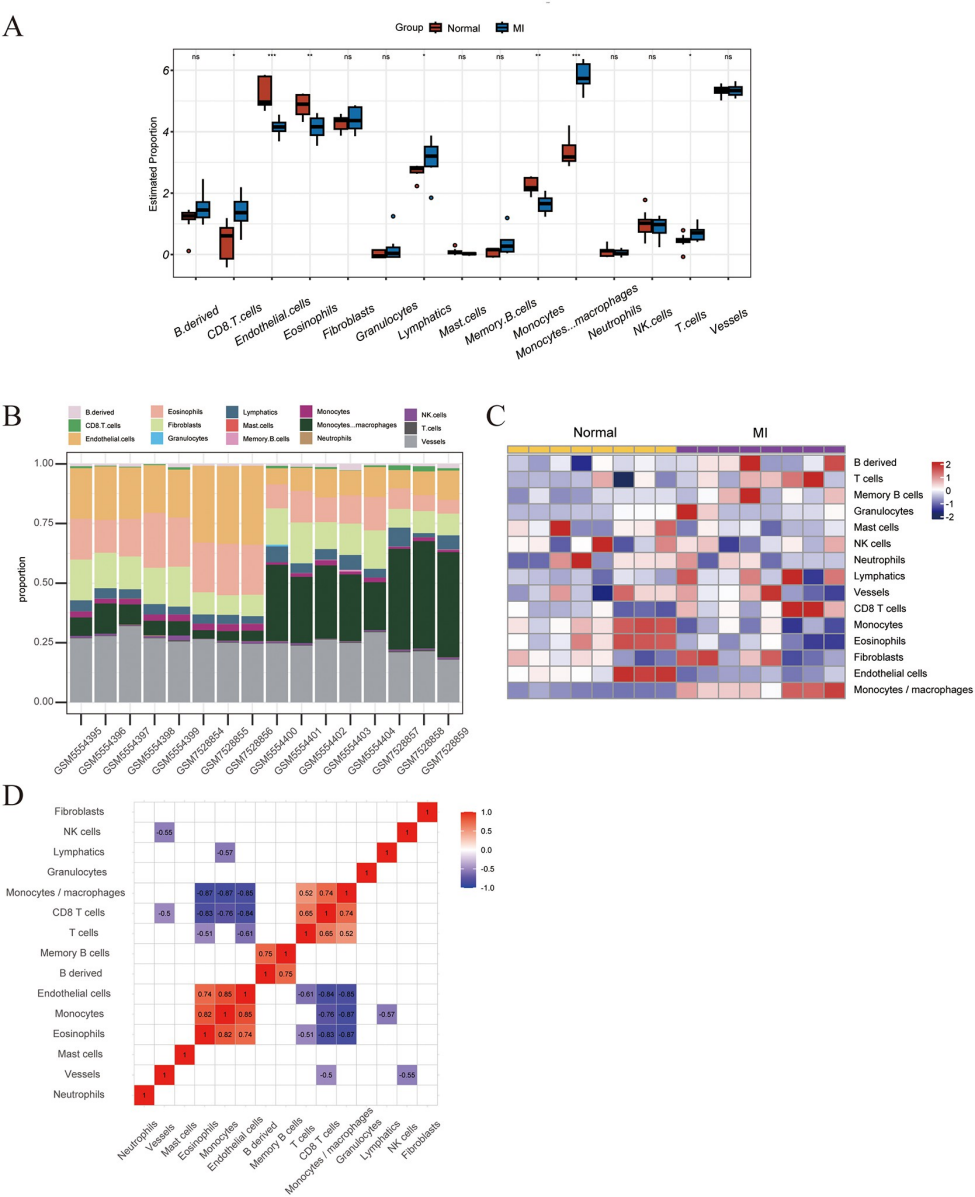
Infiltration of immune cell types compared between the MI and CON; **A**. The violin plot of the immune cell proportions; **B**. Stacked bar chart of the immune cell; **C**. Heatmap of the proportions of immune cell types; **D**. The correlation matrix of immune cell proportions.

Immune cell infiltration was analyzed using the mMCP-counter software package for seven immune cell types, including B derived cells, Granulocytes, Lymphatics, Memory B cells, NK cells, and T cells, and the MI and CON groups were compared in the combined dataset. The difference in infiltration of seven immune cell types in myocardial tissues between the MI group and the CON group was significant (P < 0.05). Specifically, CD8^+^ T cells, lymphatics, monocytes, macrophage, T cells were more abundant in the MI group. Conversely, endothelial cells, eosinophils, and monocytes were more abundant in the CON group (Fig 6A–6D).

### 3.6 Experimental validations of hub MitoDEGs expression in MI mice

**Fig 7:**
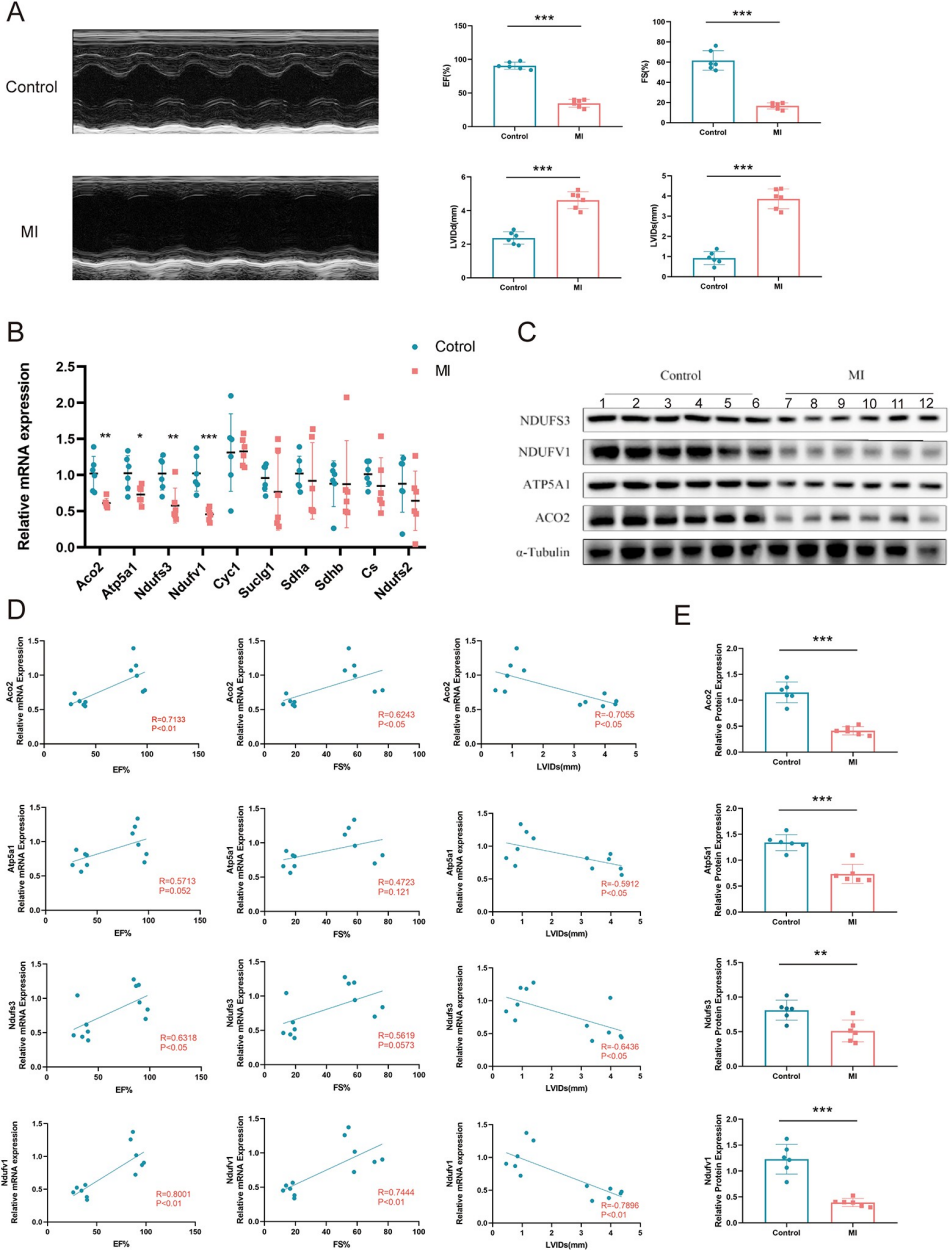
Confirmation of hub MitoDEGs expression and association with cardiac function in MI mice. **A**. Representative ultrasound images of the infarcted mouse; **B**. Hub MitoDEGs mRNA expression of CON and MI mice; **C**. Protein levels of Hub MitoDEGs by western blotting; **D**. Correlations between Aco2, Atp5a1, Ndufs3, Ndufv1 mRNA levels and cardiac functional parameters in CON and MI mice; **E**. Quantitative analysis of western blotting in cardiac tissues, Numbers represent 6 independent samples in each group. All data are expressed as the means ± SD. *P<0.05, **P<0.01, ***P<0.001 vs the CON group.

We constructed a mouse model of myocardial infarction and evaluated mouse modeling by echocardiography one week after establishing the model. M-mode echocardiography shows restricted anterior and posterior wall motion, wall thinning and left ventricular dilatation in mice. Compared to the CON group, the ejection fraction was significantly lower in the myocardial infarction group (EF 90.7±5.25 vs 34.8±5.82%, P<0.001). Mice with myocardial infarction have significantly enhanced left ventricular dilatation (LVIDd 0.92±3.12 vs 3.86±0.49mm, P<0.001) (Fig 7A). Ventricular expression of 10 central MitoDEGs (Aco2, Atp5a1, Ndufs3, Ndufv1, Cyc1, Suclg1, Sdha, Sdhb, Cs, and Ndufs2) was verified in a mouse model of myocardial infarction using PCR. Aco2, Atp5a1, Ndufs3, and Ndufv1 were all significantly lower in the MI group compared with the CON group (P < 0.05), whereas no significant differences were observed in the other MitoDEGs (Fig 7B). On this basis, the expression of Aco2, Atp5a1, Ndufs3 and Ndufv1 proteins in the MI and Control groups was further verified by Western blotting. The results showed that the protein expression levels were consistent with those of mRNA (P < 0.05) (Fig 7C). We further analyzed the correlation of the four central MitoDEGs (Aco2, Atp5a1, Ndufs3 and Ndufv1), which were significantly differentially expressed between the MI and CON groups, with EF%, FS% and LVIDd (Fig 7A). The gene expression for Aco2 and Ndufv1 was significantly positively correlated with EF% (Aco2 r = 0.7133, P<0.01; Ndufv1 r = 0.8001, P<0.01) and FS% (Aco2 r = 0.6243, P<0.05; Ndufv1 r = 0.7444, P<0.01), but highly significantly negatively correlated with LVIDd (Aco2 r = -0.7055, P<0.05; Ndufv1 r = -0.7896, P<0.01). At the same time, Atp5a1 and Ndufs3 were significantly negatively correlated with LVIDd (Atp5a1 r = -0.5912, P<0.05; Ndufs3 r = -0.6436 P<0.05), whereas Ndufs3 was significantly positively correlated with EF% (Ndufs3 r = 0.6318, P<0.05) (Fig 7D). Overall, the four central MitoDEGs we screened were strongly associated with decreased cardiac function after myocardial infarction.

## 4. Discussion

Cardiovascular disease is one of the most important diseases causing death worldwide [39]. Acute myocardial infarction is usually accompanied by obstruction of the coronary arteries, leading to a reduction in the blood flowing to the heart, which in turn affects the normal pumping function of the heart, and ultimately leads to undesirable remodeling of the heart, heart failure and even death [40, 41]. Mitochondria, as important organelles in eukaryotic cells, are involved in normal cellular metabolism and are capable of producing sufficient energy to maintain cellular homeostasis [42, 43]. Abnormal mitochondrial function in the heart will lead to a decrease in ATP production, an increase in reactive oxygen species, and programmed cell death [44].

Specific mechanistic studies between myocardial infarction and mitochondrial metabolic imbalance are lacking and need to be further explored. After analyzing the GEO dataset GSE 775 with bioinformatics methods, we discovered that the most DEGs appear one week after myocardial infarction. According to our further analysis of another two datasets, DEGs were identified to be enriched in pathways associated with mitochondrial metabolism, inflammation and immune infiltration. Therefore, analyzing the key role of mitochondria in myocardial infarction and its possible targets may help us to better understand the relationship between mitochondria and MI.

Through our screening, we found that DEGs were most notable at 1 week after MI. In fact, this is also because 1 week is the critical phase of MI inflammatory phase over to the repair phase. The first three days of MI is a period of high inflammation and whether or not it can be successfully over to the repair phase is a key factor in determining the success of the repair [45]. Besides this, a total of 4 key genes were identified, Aco2, Atp5a1, Ndufs3 and Ndufv1, which were consistent with the expression trend of the mouse myocardial infarction model we constructed. In MI, the expression levels of Aco2, Atp5a1, Ndufs3 and Ndufv1 were significantly decreased and correlated positively with reduced cardiac function.

Normal mitochondrial function and the integrity of the mitochondrial respiratory chain are important for the energy delivery to organs [46]. Ndufv1 is a nuclear-encoded structural subunit of mitochondrial respiratory chain complex I. Timely regulation after myocardial ischemia/reperfusion upregulates its expression level [47, 48]. And overexpression of Ndufv1 showed beneficial effects on ischemia/reperfusion-induced renal injury by improving mitochondrial integrity and function, which results in a reduction of oxidative stress and apoptosis, as well as a decrease in serum creatinine and blood urea nitrogen (BUN) [49]. Ndufs3 is likewise the catalytic core of mitochondrial complex I, which some studies have shown to be associated with atherosclerosis and chronic stress [50]. It is inextricably linked to mitochondrial metabolism, and its dysregulated expression will alter the mitochondrial microenvironment, which may lead to musculoskeletal senescence and affect the normal function of the organism [51]. The relationship between Ndufs3 and MI is not clear, and our study suggests a new idea that decreased expression of Ndufs3 after MI as a key component of mitochondrial complex I represents disruption of mitochondrial function, which is detrimental to cardiac repair. Another mitochondrial complex V is a key link in ATP production, and Atp5a1 is a key component of complex V, the expression of which naturally correlates significantly with OXHPOS [52]. Knockdown of Atp5a1 would severely affect cardiac function after myocardial infarction, possibly from disruption of mitochondrial homeostasis, which is also consistent with the findings of our study [53]. Additionally, it has been studied in mitochondria, and down-regulation of Atp5a1 by the use of small interfering RNAs impairs mitochondrial function and induces activation of NOD-like receptor thermal protein domain associated protein 3 (NLRP3) inflammasome, which has been demonstrated in the pathogenesis of cardiomyopathy [54]. Apart from the mitochondrial complex, we have discovered Aco2, a key enzyme of the mitochondrial tricarboxylic acid cycle, which plays a key role in cellular energy metabolism, oxidative stress and mtDNA integrity [55]. Maintenance of Aco expression favors protection of cardiac function after ischemia-reperfusion [56]. Nevertheless, the direct relationship with myocardial infarction is not clear, and our study provides evidence of this.

Mitochondrial metabolism and immune status are also closely related [57], and myocardial infarction is often accompanied by altered immune status [58]. Inflammatory response and immune cells play an important role in myocardial infarction [59]. In our study we analyzed immune cell infiltration in myocardial infarction by using the ImmuCellAI algorithm and found that T cells and CD8^+^ T cells were significantly elevated in the myocardial infarction group, which is in line with the findings of another study [60]. Besides, recruitment of the cardiac monocyte and macrophage system in myocardial infarction is also critical, as the amount of cardiac monocytes and macrophages increases rapidly in the days after myocardial infarction and does not return to the baseline level until 2 weeks later [61]. Whereas the structural integrity of mitochondria and the homeostasis of mitochondrial biogenesis favor the attenuation of macrophage infiltration after myocardial infarction and resistance to myocardial injury [62]. Mitochondria-associated enzyme activity also affects cardiac function after MI by influencing the formation of neutrophil extracellular traps [63]. Even studies on mtDNA suggest that splenic monocytes may mediate the inflammatory response after MI by affecting mtDNA [64].

In this study, we analyzed a dataset of acute myocardial infarction and determined that 1 week after myocardial infarction was the time point at which the differential gene was most pronounced in the mouse cardiac, and found that there was a crosstalk between mitochondrial autophagy and myocardial infarction, and the discovery of genes Aco2, Atp5a1, Ndufs3 and Ndufv1 provides a potential molecular target for a possible link between myocardial infarction and mitochondrial metabolism. We validated the relevant molecular targets by constructing an animal model of myocardial infarction in mice, and the molecular biology results supported our data analysis. Our study has some limitations. Firstly, there is a lack of clinical data support. Secondly, we did not perform further experiments to verify the exact effects of mitochondrial genes on the immune microenvironment, and more in vitro and in vivo experiments are needed to further support our hypothesis, which will be the main focus of our subsequent studies.

## 5. Conclusion

In summary, we identified DEGs associated with mitochondrial metabolism in acute myocardial infarction and differences in the degree of immune cell infiltration in tissues after myocardial infarction through bioinformatics studies, and screened and verified the Aco2, Atp5a1, Ndufs3 and Ndufv1 genes in in vivo experiments, suggesting that these genes are important genes associated with mitochondrial metabolism in acute myocardial infarction, which provides support for the future use of targeting mitochondrial metabolism as a direction to counteract deterioration of cardiac function after MI.

## Supporting information

S1 Fig Merged dataset with analysis of variance and visualization after removal of batch effects for GSE183272 and GSE236374. A. PCA plot before elimination of batch effect; B. PCA plot after elimination of batch effect. The distances of the sample point clusters indicate that they are from different batches and sequencing platforms. While in B, after eliminating the batch effect, the difference in distances between batches was reduced; C. PCA analysis of myocardial infarction samples before elimination of batch effect; D. PCA analysis of myocardial infarction samples after elimination of batch effect; E. The heat map of DEGs; F. The volcanic map of DEGs.
(TIF)

S2 Fig The results of GO and KEGG analysis in merging datasets. **A-C**. The enriched BP, GO, CC terms of up-regulated DEGs in GSE183272 and GSE236374; **D**. KEGG pathway enrichment results in GSE183272 and GSE236374; **E-G**. The enriched BP, GO, CC terms of up-regulated DEGs in GSE183272 and GSE236374; **H**. KEGG pathway enrichment results in GSE183272 and GSE236374.
(TIF)

S3 Fig Results of GSEA analyzes in MI. **A-B**. Metabolic signaling pathways in myocardial infarction; **C-D**. Immune-related signaling pathways in myocardial infarction.
(TIF)

S1 Raw images Original western blot images. (PDF)

## Abbreviations

Aco2: Aconitase 2
ATP: adenosine triphosphate
Atp5a1: ATP synthase F1 subunit alpha
BP: biological process
BUN: blood urea nitrogen
CC: cellular component
DEGs: Differentially expressed genes
EPC: Edge Percolated Component
GEO: Gene Expression Omnibus
GO: Gene Ontology
GPD2: glycerol-3-phosphate dehydrogenase 2
GPL81: GEO platforms 81
GSEA: Gene set enrichment analysis
IL-18: interleukin-18
IL-1β: interleukin-1β
IR: ischemia-reperfusion
KEGG: Kyoto Encyclopedia of Genomes
LVEF: left ventricular ejection fraction
MCC: Maximum Clique Centrality
MF: molecular function
MI: Myocardial infarction
MitoDEG: Mitochondria-associated DEGs
MNC: Maximum Neighborhood Component
mtDNA: mitochondrial DNA
mtROS: mitochondrial ROS
NCBI: National Center for Biotechnology Information
Ndufs3: NADH: ubiquinone oxidoreductase core subunit S3
Ndufv1: NADH: ubiquinone oxidoreductase core subunit V1
NLR: Nod-like receptor
NLRP3: NLR family pyrin domain containing 3
OXPHOS: oxidative phosphorylation
PPI: Protein-protein interaction
TCA: tricarboxylic acid cycle
TOM: topological overlap matrix
TOM: topology overlap matrix
UCP2: uncoupling protein 2
WGCNA: weighted gene co-expression network analysis
