# Supplementary figures and images for: Identification of markers correlating with mitochondrial function in myocardial infarction by bioinformatics
Source: PLoS One. 2024 Dec 30;19(12):e0316463. doi: 10.1371/journal.pone.0316463 (PMC11684664; doi:10.1371/journal.pone.0316463)

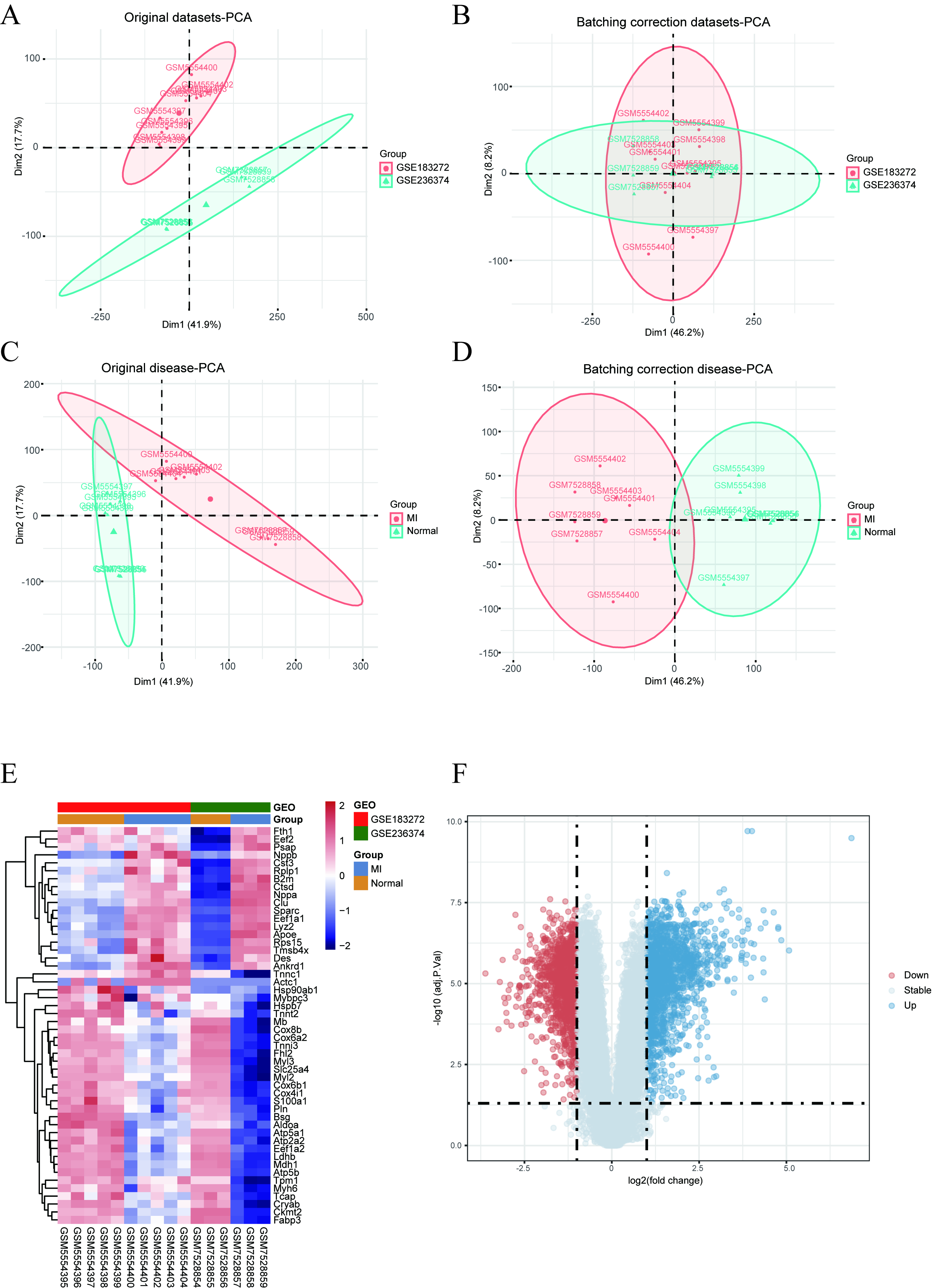

Supplement: S1 Fig — A. PCA plot before elimination of batch effect; B. PCA plot after elimination of batch effect. The distances of the sample point clusters indicate that they are from different batches and sequencing platforms. While in B, after eliminating the batch effect, the difference in distances between batches was reduced; C. PCA analysis of myocardial infarction samples before elimination of batch effect; D. PCA analysis of myocardial infarction samples after elimination of batch effect; E. The heat map of DEGs; F. The volcanic map of DEGs. (TIF) [file pone.0316463.s001.tif]

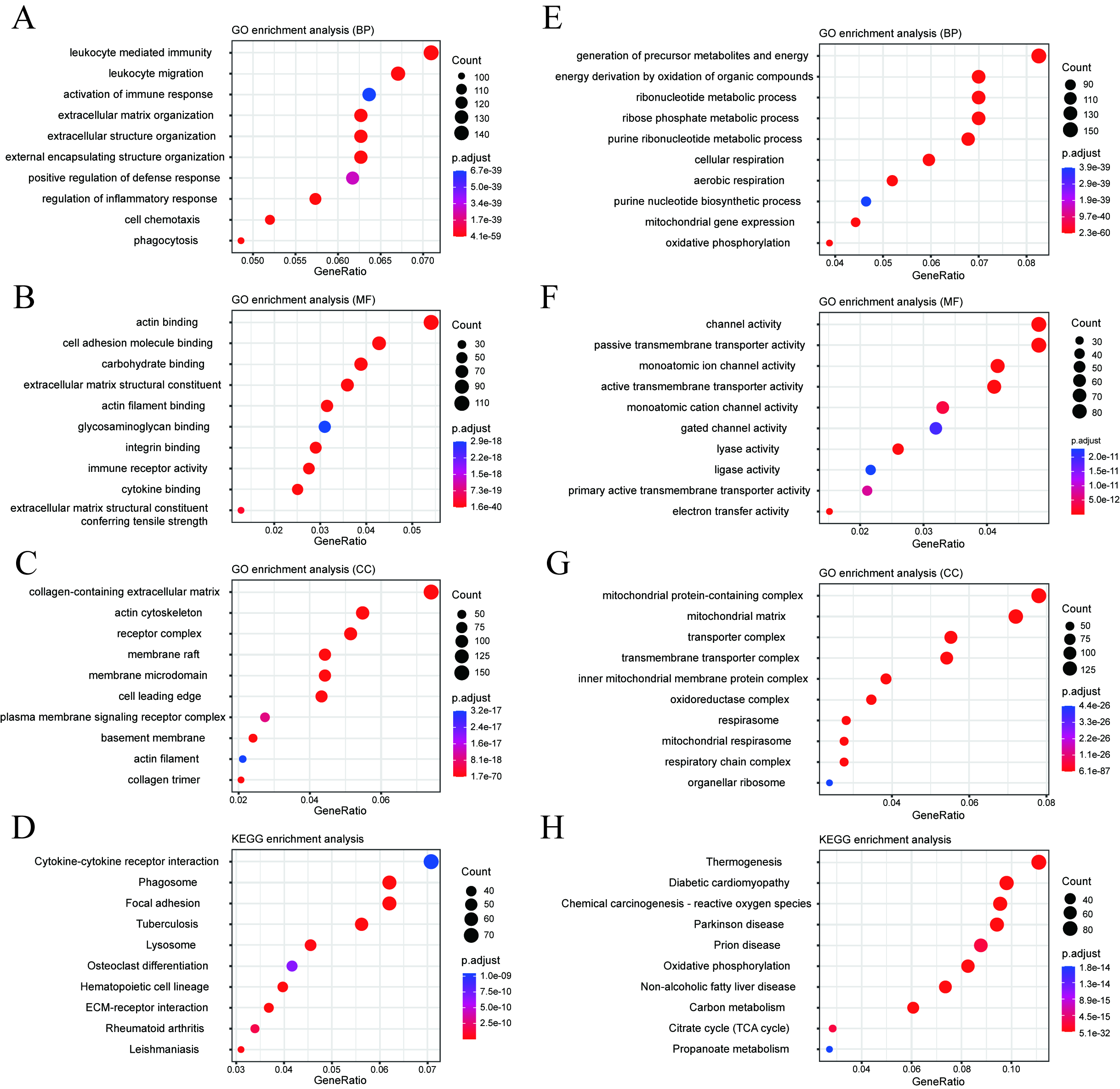

Supplement: S2 Fig — A-C. The enriched BP, GO, CC terms of up-regulated DEGs in GSE183272 and GSE236374; D. KEGG pathway enrichment results in GSE183272 and GSE236374; E-G. The enriched BP, GO, CC terms of up-regulated DEGs in GSE183272 and GSE236374; H. KEGG pathway enrichment results in GSE183272 and GSE236374. (TIF) [file pone.0316463.s002.tif]

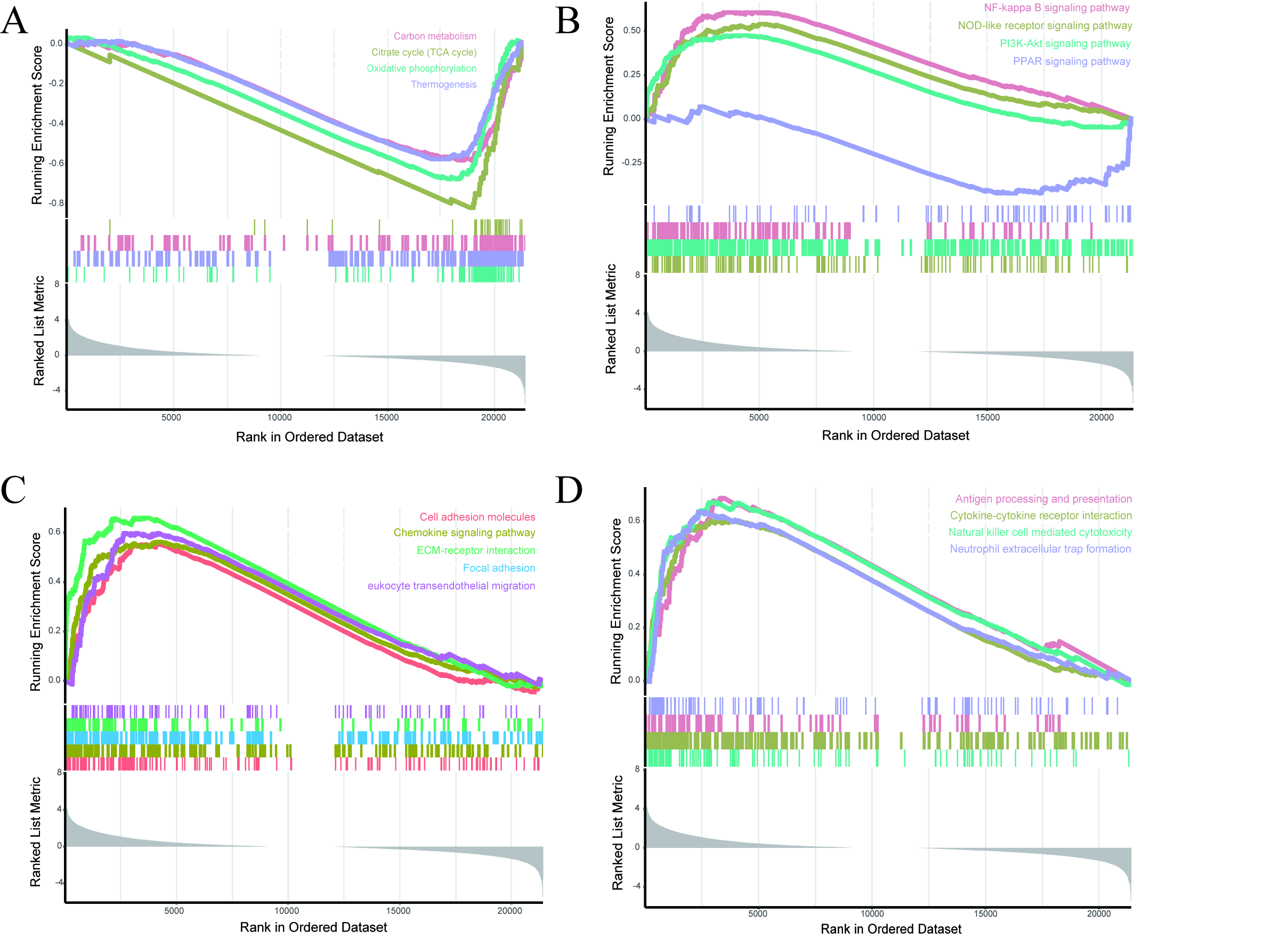

Supplement: S3 Fig — A-B. Metabolic signaling pathways in myocardial infarction; C-D. Immune-related signaling pathways in myocardial infarction. (TIF) [file pone.0316463.s003.tif]
