# Supplementary material for: Identification of markers correlating with mitochondrial function in myocardial infarction by bioinformatics
Source: PLoS One. 2024 Dec 30;19(12):e0316463. doi: 10.1371/journal.pone.0316463 (PMC11684664; doi:10.1371/journal.pone.0316463)
Supplement: S1 Raw images — (PDF) [file pone.0316463.s004.pdf]

Supporting information 2

Original western blot images

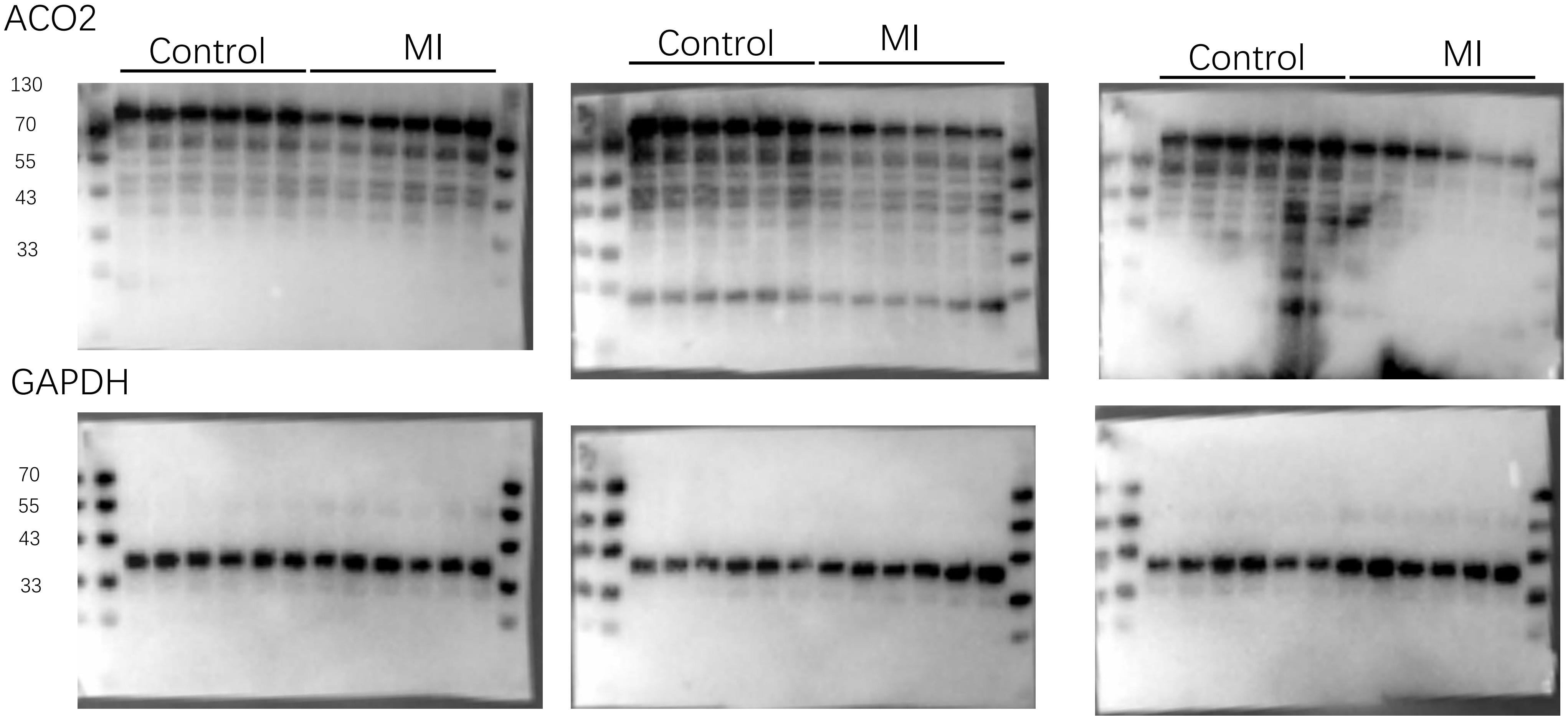
ACO2
Control
MI
Control
MI
Control
MI
130
70
55
43
33
GAPDH
70
55
43
33

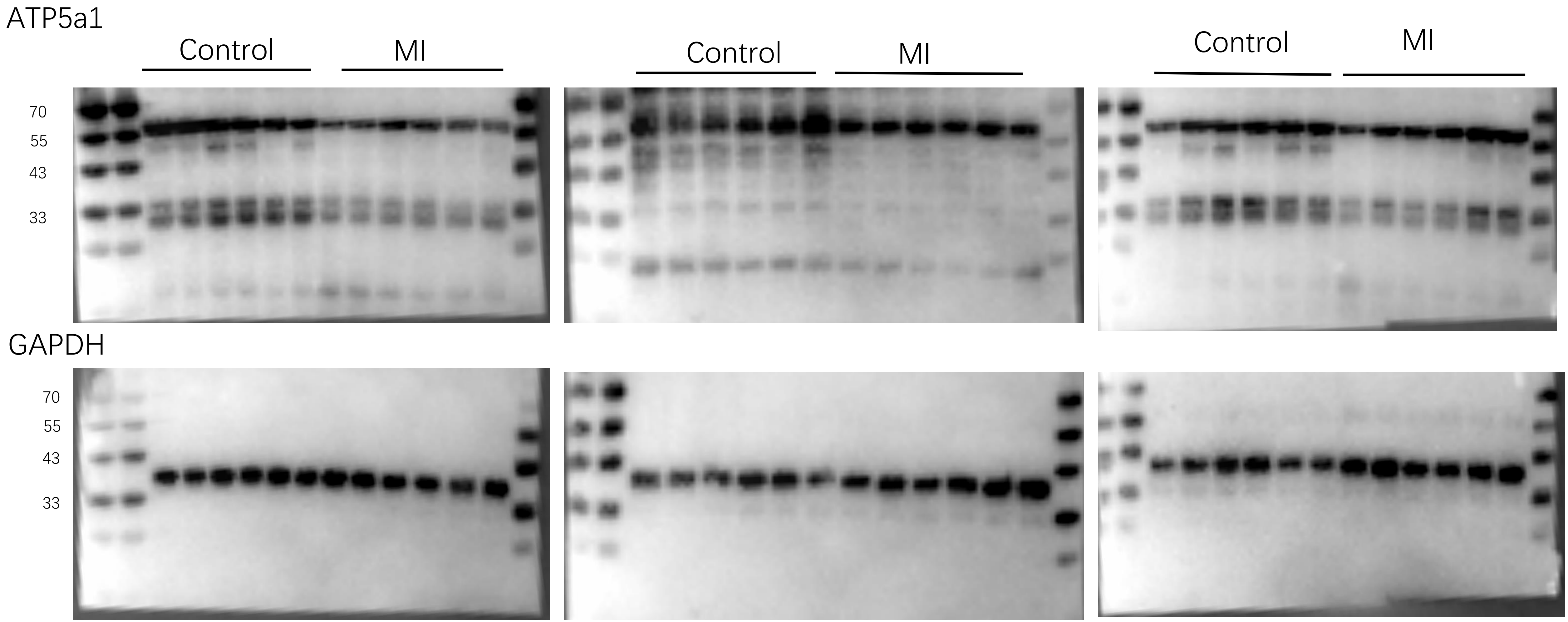
ATP5a1
Control
MI
Control
MI
Control
MI
70
55
43
33
GAPDH
70
55
43
33

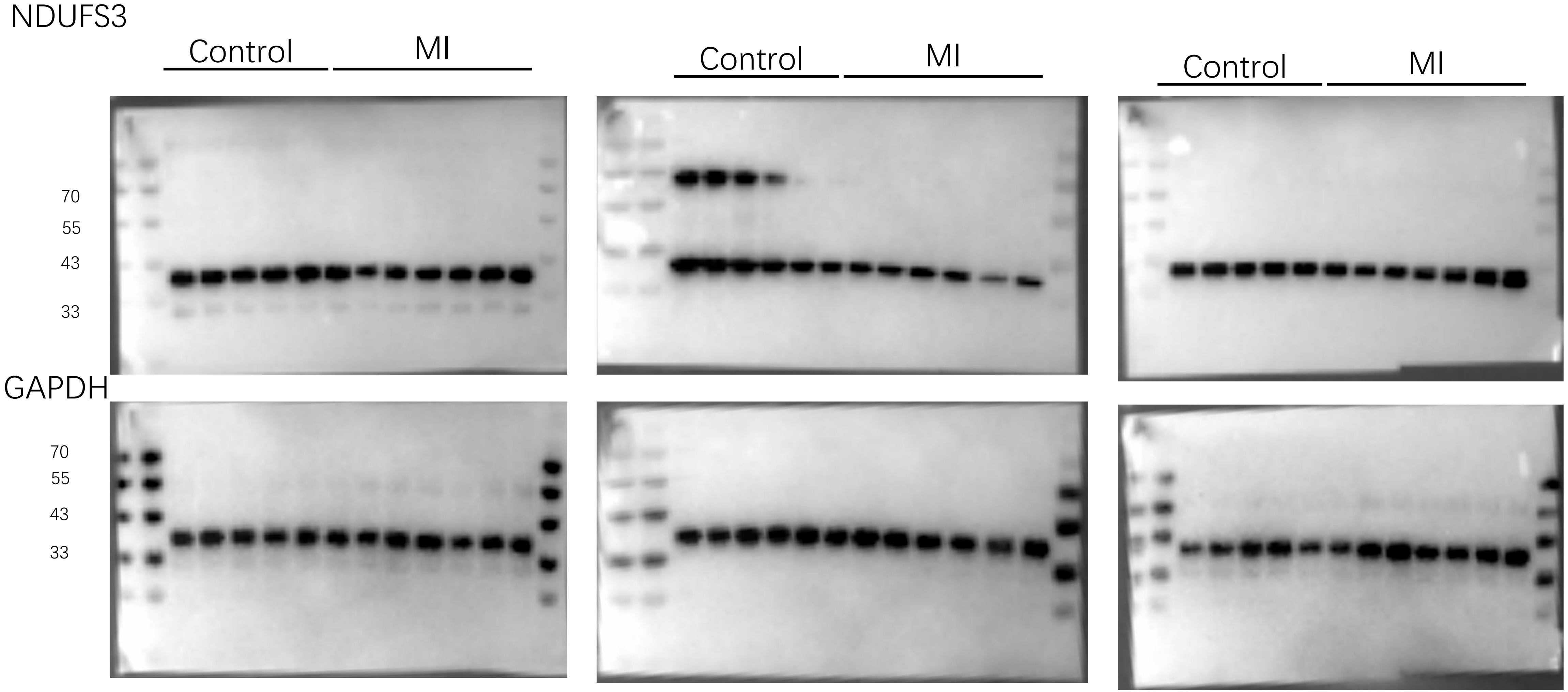
NDUFS3
Control
MI
Control
MI
Control
MI
70
55
43
33
GAPDH
70
55
43
33

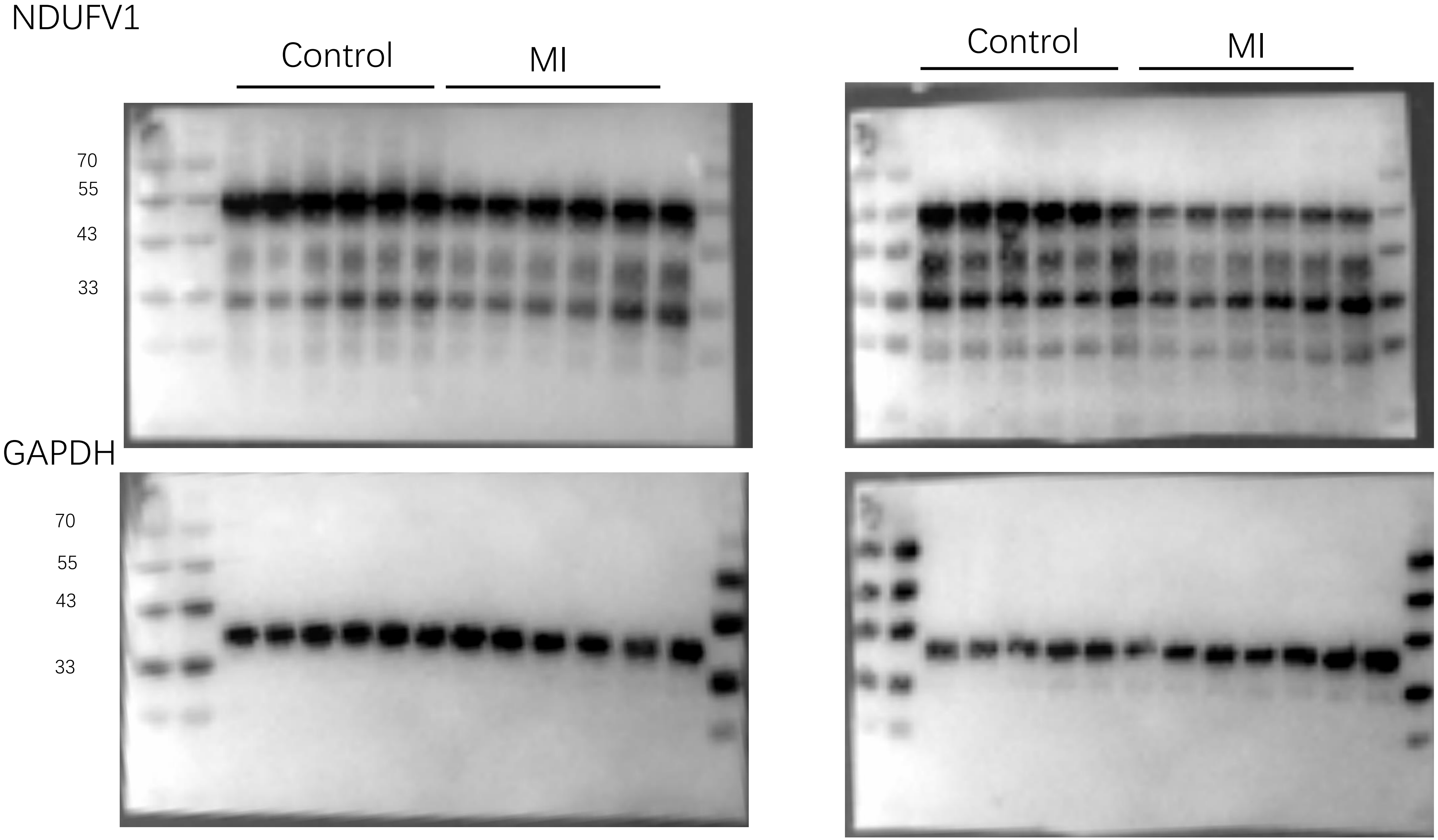
NDUFV1
Control
MI
Control
MI
70
55
43
33
GAPDH
70
55
43
33
